# Ideal Cardiovascular Health: Distribution, Determinants and Relationship with Health Status among People Living with HIV in Urban Tanzania

**DOI:** 10.5334/gh.1157

**Published:** 2022-10-12

**Authors:** Theresia A. Ottaru, Gideon P. Kwesigabo, Zeeshan Butt, Adovich S. Rivera, Pilly Chillo, Helen Siril, Lisa R. Hirschhorn, Matthew J. Feinstein, Claudia Hawkins

**Affiliations:** 1 Department of Epidemiology and Biostatistics, Muhimbili University of Health and Allied Sciences, Dar Es Salaam, Tanzania; 2 Phreesia, Inc, Delaware, US; 3 Department of Psychiatry and Behavioral Sciences, Feinberg School of Medicine, Northwestern University, Illinois, US; 4 Institute for Public Health and Management, Feinberg School of Medicine, Northwestern University, Illinois, US; 5 Department of Internal Medicine, Muhimbili University of Health and Allied Sciences, Dar Es Salaam, Tanzania; 6 Management and Development for Health, Dar es Salaam, Tanzania; 7 Department of Medical Social Sciences and Robert J Havey Institute of Global Health, Feinberg School of Medicine, Northwestern University, Chicago, Illinois, US; 8 Division of Cardiology, Department of Medicine, Feinberg School of Medicine, Northwestern University, Illinois, US; 9 Department of Medicine, Robert J Havey Institute of Global Health, Feinberg School of Medicine, Northwestern University, Chicago, Illinois, US

**Keywords:** cardiovascular health index, ideal cardiovascular health, cardiovascular diseases, HIV, Tanzania

## Abstract

**Background:**

Ageing adults living with HIV (ALHIV) have increased risk of cardiovascular diseases as a result of HIV-infection-related chronic immune activation and inflammatory responses. Cardiovascular health index (CVHI) is a valid and relatively simple index for assessing the cardiovascular health (CVH) of the general population. Use of this index among ALHIV in Sub Saharan Africa, a resource-restricted setting where it could be mostly beneficial, remains limited. Understanding of the distribution and associated factors may inform the design of optimal interventions to improve CVH of ALHIV.

**Objective:**

We aimed to assess the distribution and factors associated with CVHI scores among ALHIV in an urban setting in Tanzania.

**Methods:**

A cross-sectional study was conducted among ALHIV on antiretroviral therapy at six HIV clinics in Dar-es-Salaam, Tanzania. We summed the score of each of the seven CVHI metric to obtain the overall CVHI score and assessed the distribution of the score by sex. We then categorized the overall score into ideal (5–7), intermediate (3–4) and poor (<3) CVH categories and performed ordinal regression to identify CVHI score associated factors.

**Results:**

In all, 629 ALHIV [mean age of 43.5(SD ± 11.2) years] were enrolled. Most had ideal levels of blood glucose (96.2%) and smoking status (83.4%) while less than half had ideal BMI (48.1%), blood pressure (BP) (43.9%) and dietary intake (7.8%). Less than half (47.6%) showed ideal CVH, while less than 1% had all seven metrics at ideal level. Older age (0.96(95%CI:0.95–0.97), p-value < 0.001), being retired/unemployed (0.59(95%CI:0.43–0.81), p-value < 0.01), being employed (0.76(95%CI:0.62–0.94), p-value = 0.01) alcohol use (0.41(95%CI:0.21–0.80), p-value = 0.01) and presence of non-communicable disease comorbidities (0.68(95%CI:0.48–0.97), p-value = 0.04) had significant lower odds of ideal CVH.

**Conclusion:**

Based on our findings, interventions to improve CVH of ALHIV should target BP management, health education on diet for BMI control and reduction in alcohol consumption, particularly among ageing ALHIV with comorbidities.

## Introduction

Cardiovascular disease (CVD) constitutes a major public health problem both in high and low- and middle-income countries, with global deaths expected to exceed 23.6 million between 2020 and 2030 [1,2]. Among adults living with HIV (ALHIV), CVD is growing in importance. Globally, the risk of CVD in ALHIV is estimated to be 2.5-fold higher than people without HIV [3] due to a combination of HIV-related and behavioral risk factors [4,5,6]. Widespread access to effective antiretroviral therapy (ART) has dramatically improved the life expectancy of adults living with HIV (ALHIV). As a consequence, HIV has placed an ageing population of ALHIV at risk for CVD-related morbidity and mortality as they are living longer [7,8].

Over 25.5 million adults live with HIV in Sub-Saharan Africa (SSA), accounting for 67.7% of adult infections globally [9,10]. Few studies to date have examined the burden of CVD and associated risks in ALHIV in SSA. Among persons without HIV in Tanzania, the incidence of CVD deaths increased from **9 to 13%** between 2012 and 2016 [11], related to hypertension, diabetes and dyslipidemia as well as behavioral and dietary risks [12]. Studies examining cardiovascular health (CVH), the burden of CVD and associated risk factors among ALHIV in this setting are therefore of paramount importance [13].

Ideal cardiovascular health index (CVHI) is a measure of general CVH developed by the American Heart Association (AHA) to assess the CVH of the global general population [14]. CVHI is based on ideal levels of seven metrics; three CVD risk factors (blood pressure [BP], blood glucose and total cholesterol) and four health behaviors (smoking, physical activity, diet and body mass index [BMI]) [14]. CVHI is a valid, scalable and relatively simple index for assessing CVH [15,16].

Only a few studies have reported on CVH using CVHI score in SSA [15,16,17,18]. Among ALHIV, data on CVH assessed using CVHI is scarce. Feinstein *et al.*, assessed the distribution of the CVHI score and the difference in the scores between persons with and without HIV in rural Uganda [15]. ALHIV had better CVH compared to those without HIV, had higher mean CVHI score (4.9(SD ± 1.1) versus 4.3(SD ± 1.2)) and had more CVHI metrics at ideal levels. A significant association between CVHI score and common carotid intima media thickness (CCIMT) found in this study further suggested that CVHI is a valid measure of CVH in this region.

Beyond its utility in monitoring CVH, understanding of the distribution and associated factors could help guide the design of interventions to improve CVH and prevent development of CVD risk factors and CVD mortality among ALHIV [14]. However, the use of this index in assessing CVH of ALHIV and identifying potential targets for interventions in SSA, a resource-poor setting where it could be mostly beneficial remains limited. Therefore, the goal of the current study was to assess distribution of CVHI among ALHIV on ART, in an urban setting in Tanzania, and assess factors associated with CVHI score to identify potential targets for interventions to reduce CVD risk and improve CVH in this population.

## Materials and methods

### Study design, setting and participants

We conducted a cross-sectional study of ALHIV in six HIV care and treatment clinics (CTCs) in Dar es Salaam, Tanzania. Participants were recruited between November 2020 and January 2021. HIV CTCs were randomly selected from a list of HIV CTCs with a high volume of clients (≥1500) representing five districts in urban Dar-es-Salaam (n = 32).

Eligible participants were ALHIV (≥18 years) on ART and in care for ≥12 months prior to the start of the study. Participants who were pregnant or unable to give informed consent were excluded. Participants were selected for inclusion through systematic random sampling whereby research assistants asked every third client attending clinic whether they were interested in participating in the study.

In Tanzania, HIV care and treatment is provided free of charge under the test and treat approach [19]. Tenofovir disoproxil fumarate+Lamivudine+Dolutegravir (TDF+3TC+DTG) is the current recommended first-line regimen for ALHIV. Other regimens are recommended according to Tanzania HIV National guidelines [20]. Patients are followed monthly at clinic visits or every six months if stable (defined as being on ART for at least six months with no adverse reactions, no opportunistic infections or any other uncontrolled comorbidity but with good adherence and undetectable HIV viral loads).

Other non-ART medications, including medications for hypertension, diabetes and dyslipidemia are provided free of charge when they are available at the facility. Otherwise, patients are given a prescription to procure (out of pocket payment or covered by health insurance) the medication at a nearby pharmacy [20]. According to the Tanzania HIV National Guidelines [20], patients should receive screening for CVD risk factors including BP, body weight and height (for BMI) measurement at each clinic visit, as well as health education on lifestyle modification to reduce CVD risk. If available, the guideline also recommends blood glucose, lipids and chemistry testing every 3 months-annually.

### Data collection

A structured questionnaire collecting data on age, sex and other demographics, health insurance status, years lived with HIV infection, duration on ART, ART adherence, behavioral risk factors for CVD, family history of CVD, history of non-communicable diseases (NCD) comorbidities including CVD, medication type for chronic diseases, and perception of health status was administered to each study participant.

Health insurance was assessed with the question ‘Are you covered by health insurance or some other kind of healthcare plan?’. To assess ART adherence we used a Center for Adherence Support Evaluation (CASE) adherence index, a composite measure of self-reported ART adherence that has been validated in Tanzania ALHIV [21,22]. Behavioral risk factors such as diet, physical activity, smoking status and alcohol use followed the Swahili translated World Health Organization’s STEPS NCD risk assessment [23]. Participants who reported ever being told by a doctor or other health professional that they had hypertension, diabetes, dyslipidemia or kidney disease as well as heart attack and stroke were considered to have a history of NCD comorbidities. Data on the use of any medication or participation in lifestyle modifications as a result of these chronic conditions was also collected.

Study participants also had anthropometric measurements including height/weight and blood pressure measurement and blood was collected for non-fasting lipid profile. Random blood glucose (RBG) level was measured at the clinic with an FDA-approved, Accu-Check Guide glucose monitor by a trained nurse. Total cholesterol was analyzed using Cobas 400 analyzer (Roche Diagnostics). Portable battery-operated automatic BP cuff (Lifesource UA-767 PV) was used for BP measurement. BP measurement included three values taken at least five minutes apart and the three measurements were averaged to determine the final value. Participants who were found to have raised blood pressure, blood sugar or total cholesterol were referred to the outpatient clinic of the internal medicine department at the respective health facility for management.

For each participant, we measured body weight and height to determine BMI using standard height/weight measuring devices (stadiometer and digital scale), which were regularly calibrated to ensure accuracy. We also queried participants’ medical records to obtain data on current ART regimen type and HIV viral load (within six months of the study visit).

### Measurements

#### Definitions of ideal CVH

Each of the seven CVH metrics was evaluated and categorized as being at ideal (1 point), intermediate (0 point) of poor (0 point) according to the AHA standard guideline and previously published cutoff values [24,25]. (Table 1) RBG cut-off values instead of fasting blood glucose (FBG) were used to assess ideal blood glucose metric. Participants in this study were randomly recruited, and some were in a non-fasting state. Ideal blood glucose was defined as having RBG value of <7.8 mmol/L as used in the published literature [26].

**Table 1:** Definitions of CVHI metrics.


	POOR CVH	INTERMEDIATE CVH	IDEAL CVH

Smoking	Current smoker or have quit ≤12 months ago	Have quit >12 months ago	Never smoked

BMI	≥30 kg/m^2^	25.0–29.9 kg/m^2^	<25 kg/m^2^

Physical activity	None	1–149 min per week of moderate intensity activity and/or 1–74 min per week of vigorous intensity physical activity	≥150 min per week of moderate intensity activity and/or ≥75 min per week of vigorous intensity physical activity

Diet*	None—total servings of fruits and vegetables weekly as the cutoff for ideal intake	1–20 total servings of fruits and vegetables weekly as the cutoff for ideal intake	≥20 total servings of fruits and/or vegetables weekly as the cutoff for ideal intake

Blood pressure	SBP ≥ 140 mmHg and/or DBP ≥ 90 mmHg	SBP 120–139 mmHg or DBP 80–89 mmHg and/or on treatment for antihypertensive	SBP < 120 mmHg and DBP < 80 mmHg

Blood glucose (RBG)	≥11.1mmol/L	7.8–11 mmol/L	<7.8 mmol/L

Blood cholesterol	≥6mmol/L	5–5.9 mmol/L	<5mmol/L


BMI, body mass index; SBP, systolic blood pressure; DBP, diastolic blood pressure; * Fruit and vegetables intake.

Ideal diet was adapted from a standard definition of dietary score. As done previously [15], we used fruit and vegetable intake as a proxy for diet score (with >20 total servings of fruits and vegetables weekly as the cutoff for ideal intake) because previous studies have demonstrated associations between high fruit and vegetable intake and decreased CVD risk [27,28].

We determined the proportion of participants with ideal levels (1 point) for each metric and “ideal CVH” defined as having 5–7 metrics at ideal state. We summed the scores to obtain the overall score and determined the proportion of participants meeting none and up-to seven metrics at ideal state.

#### Health status perception

To assess health status we used the general health perception domain of the Short Form (SF) 36-Item Health Survey (Version 1.0) [29]. This tool has been previously validated and used in Tanzania to assess quality of life among individuals living with HIV and among diabetic adults [30,31]. General health domain is composed of four questions about self-rated health rated from ‘1: definitely false’ to ‘5: definitely true’. We included an additional item on general perception of ones’ health rated from ‘1: poor’ to ‘5: excellent’. Scores of each question were summed to obtain a total score with possible overall score ranging from 5–25 and used in linear regression analysis as a continuous variable

### Data analysis

Data analysis was conducted using STATA version 14 (STATA Corp Inc., TX, USA). Numerical data was presented as mean and standard deviation (median and interquartile range) while categorical variables were presented as frequencies and percentages. Chi-square test (Fisher’s exact test) and student t-test were used to compare the distribution of the CVHI metrics and overall score by sex. The overall score was then categorized into ideal (5–7), intermediate (3–4) and poor (<3) CVH and used as an ordinal variable in the regression analysis. We conducted complete case analysis using multivariable ordinal regression model [32] to identify independent associations between CVHI score and selected independent variables (p-value < 0.25 in the bivariate analysis). To reduce clustering effect at the facility level, the variable facility type was used as a cluster variable in the model. A multivariable analysis was carried out to evaluate the association between CVHI and self-reported health status using a sequential (nested) model building approach. For each model, we reported the adjusted R^2^ values and likelihood ratio test to determine the best final model.

A total of 33.5% of the study participants had missing data for CVHI score; data on total cholesterol was missing for 28.5% of the participants due to invalid laboratory results, mislabeling or declining to have blood drawn. Blood glucose, BMI and blood pressure data was missing for 7.4%, 2.2.% and 3.3%, respectively, due to incomplete recording. The remaining CVHI metrics had complete data. Missing values were assumed to be missing at random. A missing value for CVHI score was imputed as a continuous variable using Markov Chain Monte Carlo (MCMC) procedure in STATA, which assumes a multivariate normal distribution (MVN) under the missing at random assumption [33]. We generated 10 imputed data sets using age, sex and insurance status as auxiliary variables and the above analysis was repeated after multiple imputation. All analyses were two-tailed, and the significance level was set at 5%.

## Ethical consideration

The study was approved by Muhimbili University of Health and Allied Sciences (MUHAS)-MUHAS-REC-08-2020-343, National Institute for Medical Research (NIMR)-NIMR/HQ/R8a/VOL.IX/3513 and Northwestern University (STU00214283) ethics committees. All participants were asked to provide written informed consent to participate.

## Results

### Baseline characteristics of the study population

629 ALHIV with a mean age of 43.5 years (SD ±11.2), 71% female were recruited (Table 2). Only 14.3% of the participants were covered by any form of health insurance. Median duration on ART was 5 years (IQR 3–10 years); most study participants-initiated ART within six months of HIV diagnosis. Of the total, 84% had no documented comorbidities, and most (73.6%) did not have a family history of CVD (Table 2).

**Table 2:** Characteristics of the study population.


VARIABLE	TOTAL POPULATION (N = 629)	FEMALE (n = 448, 71.2%)	MALE (n = 181, 28.8%)	p-VALUE

Age (mean, SD)	43.5 (±11.2)	42.3 (±10.5)	46.7 (±12.0)	<0.001*

Level of education (n,%) No school attended Primary education Secondary education Beyond secondary	41 (6.5) 418 (66.5) 127 (20.2) 43 (6.8)	36 (8.0) 309 (69) 81 (18.1) 22 (4.9)	5 (2.8) 109 (60.2) 46 (25.4) 21 (11.6)	0.03**

Occupation status (n,%) Retired or unemployed Self-employed Employed	87 (13.8) 429 (68.2) 113 (18)	69 (15.4) 310 (69.2) 69 (15.4)	18 (9.9) 119 (65.8) 44 (24.3)	0.07**

Relationship status (n,%) Single/never married Married/cohabiting Widowed	117 (18.6) 256 (40.7) 152 (24.2)	87 (19.4) 158 (35.3) 119 (26.6)	30 (16.6) 98 (54.1) 33 (18.2)	0.41**

Insurance status (n,%) Yes	90 (14.3)	57 (12.7)	33 (18.2)	0.07**

Facility name (n,%) Amana Sinza Temeke Mnazi Mmoja Bunju Vijibweni	110 (17.5) 121 (19.2) 115 (18.3) 135 (21.5) 66 (10.5) 82 (13.0)	84 (18.8) 84 (18.8) 79 (17.7) 97 (21.7) 52 (11.6) 52 (11.6)	26 (14.4) 37 (20.4) 36 (19.9) 38 (21) 14 (7.7) 30 (16.6)	0.19**

ART regimen (n,%) TDF+3TC+DTG (TLD) Other	559 (89) 69 (11)	396 (88.4) 52 (11.6)	163 (90.6) 17 (9.4)	0.43**

HIV viral load (n,%) ≤50 copies >50 copies	544 (86.6) 84 (13.4)	395 (88.2) 53 (11.8)	149 (82.8) 31 (17.2)	0.07**

Years lived with HIV infection (median, IQR)	6 (3–11)	6 (3–11)	5 (2–10)	0.31*

Duration on ART (median, IQR) in years	5 (3–10)	5 (3–10)	4 (2–10)	0.35*

Adherence to ART (n,%) Poor adherence (score < 10) Good adherence	77 (12.2) 552 (87.8)	57 (12.7) 391 (87.3)	20 (11.1) 161 (89)	0.56**

Alcohol use (past 30 days) (n,%)	158 (25.1)	108 (24.1)	50 (27.6)	0.34**

Prevalent comorbidities (self-reported) (n,%) Cardiovascular diseases (MI or stroke) Hypertension Diabetes Hyperlipidemia	69 (10.9) 67 (10.7) 19 (3.0) 7 (1.1)	57 (12.7) 55 (12.3) 12 (2.7) 5 (1.1)	12 (6.6) 12 (6.6) 7 (3.9) 2 (1.1)	0.03** 0.04** 0.43** 1.0***

Medication use (Hypertension, diabetes, dyslipidemia) (n,%) Any treatment Antihypertensive medication Glucose lowering medication Lipid lowering medication	31 (4.9) 22 (3.5) 10 (1.6) 2 (0.3)	24 (5.4) 16 (3.6) 7 (1.6) 2 (0.5)	7 (3.9) 6 (3.3) 3 (1.7) 0 (0.0)	0.43*

Lifestyle modification (n,%) Yes	163 (25.8)	11 (24.8)	52 (28.7)	0.31**

Family history of CVD (n,%) Yes	166 (26.4)	136 (30.4)	30 (16.6)	<.001**

Self-reported health status (medina, IQR)	20 (19–22.5)	20.3 (19–22.5)	20.6 (19–22.5)	0.40*


CVD: cardiovascular disease, SD: Standard deviation, TDF: Tenofovir, 3TC: Lamivudine, DTG: Dolutegravir, TLD: Tenofovir, Lamivudine, Dolutegravir. Self-reported health status possible range 5–25. * Student t-test, ** Chi-square test, *** Fisher’s exact test.

### Distribution of the CVHI metrics and scores

Most of the study participants with lab values had ideal levels of RBG (96.2%), with the next-highest metric being ideal smoking status (84.3%), while the lowest was ideal diet level at only 7.8%. Males were more likely to report being current and/or having quit smoking in less than 12 months before the study (poor CVH in terms of smoking) compared to females (18.8% versus 2.2%).

Less than half of the participants had ideal BMI level of <25kg/m^2^ (48.1%) and BP (43.9%). Males were more likely to be at the ideal level for BMI compared to females (55.1% vs. 45.3% p = 0.001). In contrast, more females had ideal BP level compared to males (46% vs 40%; p = 0.10). Overall mean systolic BP was 123.5 (SD ± 21.2) and was significantly higher in males vs. females (127.9 (SD ± 22.8)) vs. 121.8 (SD ± 20.3); p = 0.01). The prevalence of hypertension (defined as SBP ≥140 mmHg and/or DBP ≥90 mmHg) in our study population was 14.3%. Seventy-four percent of participants were at an ideal level for physical activity, while about 79.3% had ideal cholesterol levels (Table 3).

**Table 3:** Distribution of CVHI metrics and overall score.


VARIABLE	TOTAL POPULATION (N = 629)	FEMALE (n = 448, 71.2%)	MALE (n = 181, 28.8%)	p-VALUE

Ideal CVH metrics				

Smoking Poor (Current smoker or quit smoking ≤12 months ago) Intermediate (Have quit >12 months ago) **Ideal (not current) smoking status n (%)**	44 (7.0) 55 (8.74) **530 (84.3)**	10 (2.2) 14 (3.1) **424 (94.6)**	34 (18.8) 41 (22.7) **106 (58.6)**	<0.001*

BMI BMI (Mean, SD) Poor (≥ 30 kg/m^2^) Intermediate (25.0–29.9 kg/m^2^) **Ideal BMI (<25), n (%)**	27.3 (±17.9) 153 (24.9) 166 (26.9) **296 (48.1)**	26.6(±7.9) 130 (29.6) 110 (25.1) **199 (45.3)**	28.9 (±31) 23 (13.1) 56 (31.8) **97 (55.1)**	0.15* <0.001*

Physical activity Minutes of vigorous activity per week (mean, SD) Minutes of moderate activity per week (mean, SD) Poor Intermediate **Ideal Physical activity, n (%)**	229.4 (±618.3) 884 (±1226.2) 139 (22.1) 26 (4.13) **464 (73.8)**	126.4 (±398.9) 974 (±1261.6) 103 (23) 19 (4.2) **326 (72.8)**	484.4 (±920.2) 661.7 (±1106.1) 36 (19.9) 7 (3.9) **138 (76.24)**	<0.001* <0.01* 0.37**

Diet Servings of vegetables per week (mean, SD) Servings of fruit per week Poor Intermediate **Ideal diet, n (%)**	9.8 (±5.9) 7.3 (±6.6) 2 (0.32) 578 (91.9) **49 (7.8)**	9.9 (±6.1) 7.1 (±7.0) 1 (0.2) 416 (92.9) **31 (6.9)**	9.5 (±5.3) 7.8 (±5.4) 1 (0.6) 162 (89.5) **18 (9.9)**	0.25* 0.48* 0.51**

Cholesterol**** Total cholesterol (mean, SD) Poor Intermediate **Ideal cholesterol, n (%)**	4.3 (±0.9) 22 (4.9) 71 (15.8) **357 (79.3)**	4.4 (±0.9) 15 (4.5) 55 (16.6) **262 (78.9)**	4.2 (±1.0) 7 (5.9) 16 (13.6) **95 (80.5)**	0.15* 0.54**

BP SBP (mean, SD) DBP (mean, SD) Poor Intermediate **Ideal BP, n (%)**	123.5 (±21.2) 78.9 (±15.9) 87 (14.3) 254 (41.8) **267 (43.9)**	121.8 (±20.3) 78.8 (16.8) 56 (12.8) 181 (41.5) **199 (45.6)**	127.9 (±22.8) 79.1 (±13.7) 31 (18) 73 (42.4) **68 (39.6)**	0.001* 0.84* 0.10**

Blood glucose/diabetes**** RBG (mean, SD) Poor Intermediate **Ideal glucose status, n (%)**	5.2 (±1.7) 7 (1.2) 15 (2.6) **560 (96.2)**	5.2 (±1.6) 4 (0.9) 13 (3.1) **399 (95.9)**	5.2 (±2.0) 3 (1.8) 2 (1.2) **161 (97)**	0.88* 0.40**

Overall score (mean,SD) Poor CVH (<3) Intermediate CVH (3–4) **Ideal CVH (5–7)**	4.8 (1.1) 22 (5.2) 198 (47.1) **200 (47.6)**	4.4 (1.1) 12 (3.9) 149 (48.1) **149 (48.1)**	4.3 (1.2) 10 (9.09) 49 (44.6) **51 (46.4)**	0.25* 0.10**


BMI: kg/m^2^, SBP: Systolic blood pressure (mmHg), DBP: Diastolic blood pressure (mmHg), Total cholesterol (mmol/L), RBG: Random Blood Glucose (mmol/L). * Student t-test, ** Chi-square test, *** Fisher’s exact test. **** Missing values: 28.5% for cholesterol levels, 7.4% for blood glucose levels, 3.3% for BP levels, 2.2% for BMI measurements.

The distribution of overall ideal CVH metric by sex is summarized in Figure 1 (Supplementary file 1). Mean CVHI score was 4.4 (SD ± 1.1), with no significant difference between females and males (p-value = 0.25). Less than 1% of the study population had ideal levels of all seven CVHI metrics. Less than half of the study population (47.6%) were categorized as having ‘ideal CVH’ with 5–7 CVHI metrics at ideal levels. Excluding the ideal diet variable, where only 7.8% of the participants were at ideal state, the prevalence of meeting all the CVHI metrics improved from less than 1% to 14.76%.

### Determinants of ideal CVH

In multivariable analysis assessing factors associated with overall CVH, a one-year increase in age was significantly associated with 0.96 lower odds of ideal CVH (95% CI 0.95 to 0.97, p-value < 0.001) (Table 4). Alcohol users and participants with comorbidities such as hypertension, diabetes and dyslipidemia had significantly lower odds of ideal CVH. A one-year increase in the duration of ART use was associated with two percent higher odds of ideal CVH; however, these results were not statistically significant after multivariable analysis. Results of multivariable regression analysis after multiple imputation showed that there is significant association between age and alcohol consumption and CVHI score after adjusting for presence of comorbidities and medication for NCD comorbidities. A one-year increase in age was associated with a significant 3% lower odds of ideal CVH (95% CI 0.96 to 0.99, p-value < 0.001) while alcohol use was associated with 0.46 lower odds of ideal CVH (95% CI 0.30 to 0.73, p-values = 0.001). Table 1, Supplementary file 2.

**Table 4:** Determinants of ideal CVH.


	BIVARIATE MODELS		MULTIVARIABLE MODELS	
	
	COR (95% CI)	p-VALUE	AOR (95% CI)	p-VALUE

Age	0.97 (0.95–0.98)	<0.001	0.96 (0.95–0.97)	<0.001

Sex Female Male	Ref. 0.84 (0.53–1.35)	0.48	–	–

Level of education Primary education Secondary education Beyond secondary No school attended	Ref. 1.32 (0.78–2.23) 0.83 (0.28–2.42) 0.76 (0.29–1.98)	0.31 0.73 0.58	–	–

Occupation status Self-employed Retired or unemployed Employed	Ref. 0.63 (0.42–0.96) 0.81 (0.68–0.97)	0.03 0.02	Ref. 0.59 (0.43–0.81) 0.76 (0.62–0.94)	<0.01 0.01

Relationship status Married/cohabiting Single/never married Widowed	Ref. 1.28 (1.08–1.53) 0.94 (0.73–1.21)	0.01 0.66	Ref. 1.07 (0.91–1.27) 1.03 (0.85–1.25)	0.41 0.71

Insurance status No Yes	Ref. 0.66 (0.37–1.17)	0.16	Ref 0.85 (0.44–1.66)	0.64

ART regimen TDF+3TC+DTG (TLD) Other	Ref. 0.76 (0.43–1.36)	0.36	–	–

HIV viral load ≤50 >50	Ref. 1.10 (0.76–1.60)	0.61	–	–

Years lived with HIV infection	1.02 (1.00–1.04)	0.11	1.02 (0.98–1.06)	0.27

Duration of ART (years)	1.02 (1.01–1.05)	0.09	1.01 (0.98–1.05)	0.37

Adherence to ART Good adherence Poor adherence	Ref. 0.97 (0.56–1.68)	0.92		

Alcohol use No Yes	Ref. 0.16 (0.24–0.85)	0.01	Ref. 0.41 (0.21–0.80)	0.01

Presence of comorbidities No Yes	Ref. 0.65 (0.47–0.92)	0.01	Ref. 0.68 (0.48–0.97)	0.04

NCD Medication use (HTN, diabetes, dyslipidemia) (n,%) No Yes	Ref. 0.84 (0.50–1.42)	0.53	–	–

Lifestyle modification (n,%) No Yes	Ref. 0.92 (0.60–1.40)	0.71	–	–

Family history of CVD (n,%) No Yes	Ref. 1.05 (0.64–1.71)	0.84	–	–


Lifestyle modification to control hypertension, diabetes, hyperlipidemia, obesity, obesity, smoking, diet and increase physical activity. Comorbidities include self-reports of myocardial infraction (heart attacks), stroke, hypertension, diabetes or hyperlipidemia.

### Ideal CVH and self-reported health status

In an unadjusted model, a one-unit increase in CVHI score was associated with 0.02 (95% CI -0.09 to 0.39, p-value = 0.16) higher health-status score. In adjusted models using the sequential (nested) approach, there was no significant association between CVHI score and health status. Likelihood ratio test comparing model 2 to model 3 (and model 3 and model 4) was not statistically significant. Table 2, Supplementary file 2.

## Discussion

In our analysis of health using CVHI score in a cohort of ALHIV in urban Dar-es-Salaam, fewer than 1% of study participants had all seven CVHI metrics at ideal levels. These results are consistent with other studies using a similar metric in ALHIV in SSA. In rural Uganda [15], the prevalence of meeting ideal levels of all seven CVHI metrics was 2.9% among ALHIV and <1% in rural South Africa [16], where 50% of its participants were ALHIV. Data is also similar to that from the US [34], Europe [35] and Asia [36,37], suggesting that maintaining all seven CVHI metrics at an ideal state is a hard target to achieve. Although the percent with 7 ideal metrics was low, similar to previous literature, about half of participants had 5–7 CVHI metrics at the ideal state (categorized as ideal CVH) [16].

The overall mean CVHI score of 4.8 (SD ± 1.1) reported in our study, as well as that reported in Uganda, 4.9 (SD ± 1.1) and 4.3 (SD ± 1.2), and rural South Africa 4.4 (SD ± 1.2) [15,16], was relatively higher compared to mean scores reported in the US (3.45(SD ± 0.01) and Europe (2.8).[35,38]. The difference could be attributed to the differences in the age distribution between the two settings; however, the mean ages of participants in these studies are comparable. The majority of ALHIV in Tanzania are more frequently seen in healthcare settings compared to those without HIV and, as a result, have more access to health education on healthy lifestyle and screening of HIV-related NCDs. However, most HIV CTCs in Tanzania are poorly equipped with trained providers, tools and medications to appropriately diagnose, monitor and manage patients for CVD or its associated risks; thus, it is unlikely better access among ALHIV accounts for these differences [39,40].

Among the individual CVHI metrics, the highest prevalence of ideal score was observed for RBG (96.2%) followed by being a current smoker (84.2%). During data collection participants were not asked if they had eaten before coming to the clinic nor were they requested to fast before data collection. Considering the timing of data collection (morning hours), it could be possible that the recorded value for majority of the participants was for fasting blood glucose and thus an overestimation of the proportion of participants with RBG level at an ideal state and overall CVH in this study population. Repeated analysis of blood glucose values, using cut-off value for fasting blood glucose of <5.6mmol/L [14,25] reduced the proportion of participants at ideal state for blood glucose slightly from 96.2% to 83%. Re- analysis also reduced the proportion of participants with ideal CVH from 47.6% to 44%. Results of regression analysis using fasting blood glucose results in a significant relationship between duration on ART and CVH, whereby a one-year increase on ART was associated with 1.06 higher odds of good CVH. This association was only significant in the univariate analysis when random blood glucose levels were used. However, reports from other studies similarly determined blood glucose [16,35,38] and smoking [15,18,35] as the main determinants of ideal CVH, with the majority of study populations having these metrics at an ideal state.

The main contributors to the poor CVH in our study were BP (43.9%), BMI (48.1%) and diet (7.8%); less than half of our study population had these metrics at ideal levels. Although similar metrics were reported to be drivers of poor CVH among ALHIV in Uganda [15], the proportion of participants at ideal levels was higher (66.7% for BP and 67.6% for BMI). The difference in prevalence observed could be attributed to the study setting. In Uganda, participants were recruited from a clinic with relatively well-established CVD care compared to our setting. BP is checked every visit and patients are routinely attended by a physician, offering an opportunity for frequent screening and health education on CVH. In a population without HIV, a majority of the studies considered BP and BMI as major drivers of poor score with less than half of the study population in all the studies being at ideal state for these metrics [18,35,38]. Comparing the contribution of diet to CVH demonstrated among different studies should be done with caution, because of the differences in assessing ideal diet in different settings and whether these measures are sufficient sensitive markers of CVH. A possible explanation for low prevalence of ideal diet in our study is that many people in this setting have two large starch-based meals a day due to insufficient purchasing power [41]. Although these meals often contain small portions of fruits and vegetables, in total they do not reach the recommended intake.

Previous studies have suggested ALHIV are at higher risk of CVD and related risk factors due to long term ART use and HIV-infection related to immune activation [42]. Paradoxically, in our study, duration on ART appears to have a positive association with ideal CVH although only in the univariable analysis. Studies have shown that ART initiation is associated with significant reduction in inflammation, viral replication and endothelial dysfunction [43,44,45] which could have resulted in beneficial effects on ideal CVHI score in our patients. Our study population had been on ART for a relatively short period of time (median 5 years), with a quarter of the population (n = 153) on the second year of treatment, thus probably still benefiting from positive effects of ART use on CVH. In addition, the majority of our participants were on TLD, a non-PI (Protease Inhibitor)-based ART regimen that is known to be associated with a relatively lower risk of CVD compared to older PI and NNRTI (non-nucleoside reverse transcriptase inhibitors) based regimens. [42] Participants with VL copies of >50 IU/mL were found to have ten percent higher odds of ideal CVH in the bivariate analysis, but these results were not statistically significant in the multivariable analysis.

Both in complete case analysis and the analysis after multiple imputation, age and alcohol use were independent risk factors for poor CVH among ALHIV in our setting. CVD risk increases substantially with age as a result of the age-associated changes to the heart and blood vessels [46]. Alcohol use is common among ALHIV, with reported prevalence ranging from 8% to 42% [47]. Similar to HIV-infection, alcohol is an established cause of systemic inflammation and immune activation [47]. Studies have also associated alcohol use with increased odds of high BP [48], total serum cholesterol and triglycerides levels [49]. Evidence from our study and from previous studies suggests an immediate need for interventions to advocate for healthy drinking among ALHIV. Other factors found to be independently associated with good CVH in other studies, such as level of income and level of education, were not significant in this study [15,16,18].

Although CVHI score has been shown to be associated with reduced number of physically and mentally unhealthy days in other studies [50], our study did not find a significant association between CVHI and self-reported health status. Our data on health status was highly skewed, with the majority of our study participants reporting excellent health status. This could be because our study included patients who are stable on ART—the majority had undetectable viral loads, have been living with HIV-infection for 6 median years and only ideal levels of CVD risk were being assessed. Based on self-report, only about 10% had reported actual CVD comorbidities (stroke, myocardial infraction) that might be associated with reduced quality of life.

There were several limitations to this study. First, a third of the study participants had missing data for CVHI score, the large majority of whom were missing data on total cholesterol levels due to invalid laboratory results. This could have resulted in an overestimation of ideal CVHI score. Second, self-reported data on health status used in this study is prone to desirability bias and thus a potential for underestimation of the association between CVHI score and health status that was presented. Third, a generic measure of health status (SF-36) that was used to assess health status in this study may not have been specific enough to capture small but significant changes in health status, particularly HIV/CVD-specific dimensions of health status. In addition, cross-sectional study design that was used in this study limit claims regarding temporal relationship between determinants, health status and CVH. The timing of data collection could have introduced selection bias in this study. The majority of our participants were enrolled during early hours of the day—the majority of the ALHIV preferred visiting the clinic early so that they get to go to work afterwards. Due to this, we might have included healthier clients who are health conscious thus causing an over-estimation of the prevalence of ideal CVH.

## Conclusion

In our study of urban ALHIV, almost half of the study population had ideal CVH with 5–7 metrics at ideal levels. However, less than 1% of the participants had all seven metrics at ideal levels. Efforts to further improve CVH should focus on preventing and controlling BP, continuous health education on diet for controlling BMI and reduction in alcohol use. Such efforts must be targeted at the ageing population of ALHIV with comorbidities, a sub-population found at lower odds of good CVH in our study. Future studies among larger cohorts of ALHIV should focus on the application of CVHI in monitoring CVH and determinants of change in CVH over the course of HIV-infection.

## Additional Files

The additional files for this article can be found as follows:

10.5334/gh.1157.s1 Supplementary file 1. Figure 1.

10.5334/gh.1157.s2 Supplementary file 2. Tables 1 to 3.

## Abbreviations

AHA = American Heart Association, BMI = Body Mass Index, CVD = Cardiovascular Disease, CVH= Cardiovascular Health, CVHI = Cardiovascular Health Index, DTG: Dolutegravir, HDL-C = High Density Lipoprotein Cholesterol, HIV = Human Immune Deficient Virus, HRQOL = Health related quality of life, LDL-C = Low Density Lipoprotein Cholesterol, TDF: Tenofovir Disoproxil Fumarate, 3TC: Lamivudine, TLD: Tenofovir, Lamivudine, Dolutegravir.
