# Supplementary figures and images for: Ideal Cardiovascular Health: Distribution, Determinants and Relationship with Health Status among People Living with HIV in Urban Tanzania
Source: Glob Heart. 2022 Oct 12;17(1):74. doi: 10.5334/gh.1157 (PMC9562968; doi:10.5334/gh.1157)

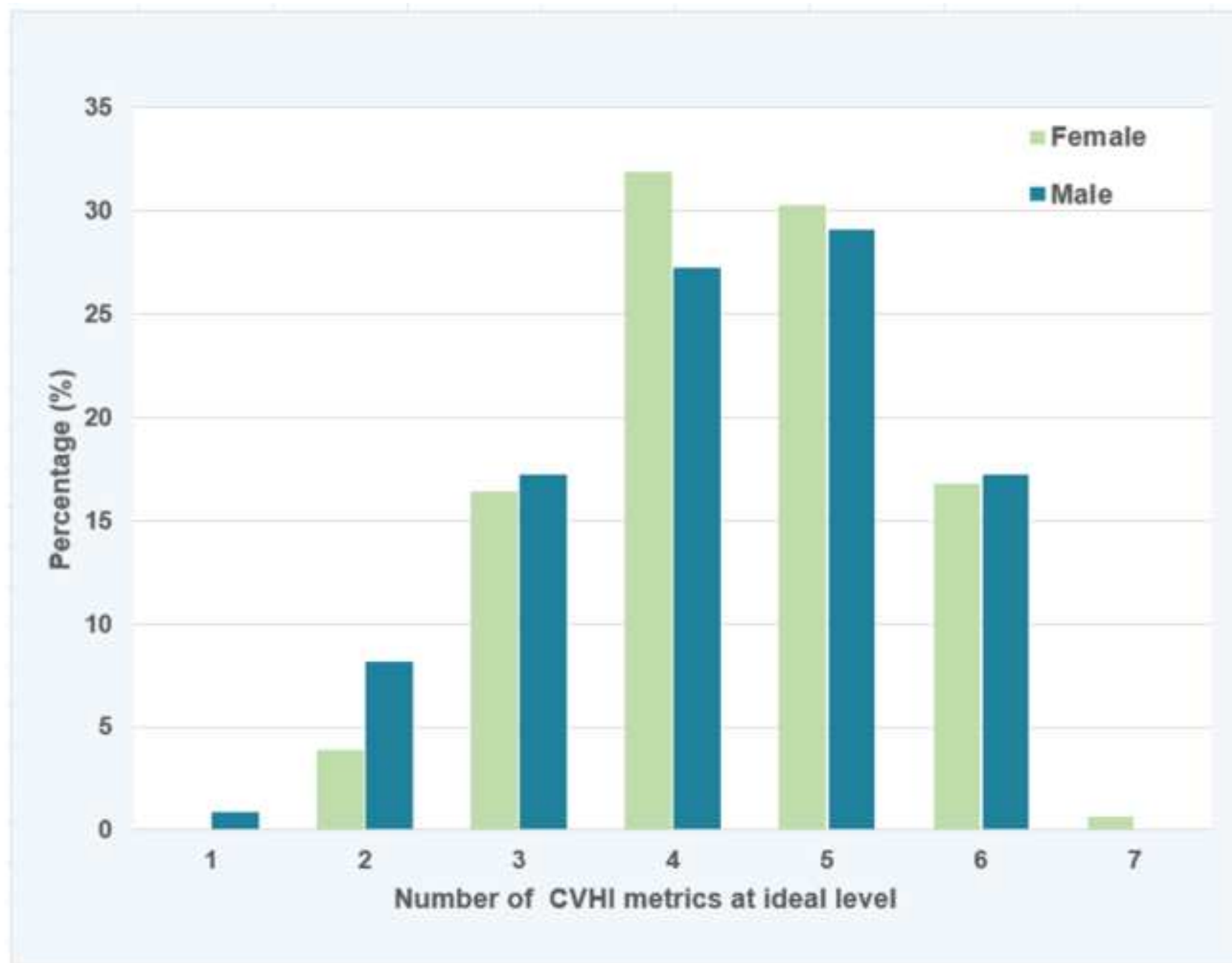

Figure 1: Distribution of the number of CVHI metrics at ideal level by sex

Supplement: Supplementary file 1. — Figure 1. [file gh-17-1-1157-s1.pdf]
