# Supplementary material for: Ideal Cardiovascular Health: Distribution, Determinants and Relationship with Health Status among People Living with HIV in Urban Tanzania
Source: Glob Heart. 2022 Oct 12;17(1):74. doi: 10.5334/gh.1157 (PMC9562968; doi:10.5334/gh.1157)
Supplement: Supplementary file 2. — Tables 1 to 3. [file gh-17-1-1157-s2.pdf]

Ideal Cardiovascular Health: distribution, determinants and relationship with health status among people living with HIV in urban Tanzania

Supplementary material

Table 1: Results of bivariate and multivariable regression after multiple imputation

	Bivariate models		Multivariable models	
	COR (95% CI)	p-value	AOR (95% CI)	p-value
Age	**0.98 (0.97 – 0.98)**	**<0.001**	**0.97 (0.96 – 0.99)**	**<0.001**
Sex				
Female	Ref.		-	-
Male	0.79 (0.49 – 1.30)	0.35		
Level of education				
Primary education	Ref.		-	-
Secondary education	1.06 (0.95 – 1.81)	0.84		
Beyond secondary	0.83 (0.36 – 1.95)	0.68		
No school attended	0.81 (0.42 – 1.56)	0.52		
Occupation status				
Self-employed	Ref.		-	-
Retired or unemployed	0.76 (0.49 – 1.20)	0.25		
Employed	0.88 (0.65 – 1.21)	0.43		
Relationship status				
Married/cohabiting	Ref.		-	
Single/never married	1.17 (0.80 – 1.71)	0.41		
Widowed	0.92 (0.59 – 1.42)	0.70		
Insurance status				
No	Ref.		Ref	
Yes	**0.68 (0.38 – 1.20)**	**0.18**	0.78 (0.40 – 1.53)	0.47
ART regimen				
TDF+3TC+DTG (TLD)	Ref.		-	-
Other	0.90 (0.56 – 1.44)	0.67		
HIV viral load				
≤50	Ref.		-	-
>50	1.02 (0.62 – 1.54)	0.92		
Years lived with HIV infection	1.01 (0.99 – 1.04)	0.30	-	-
Duration on ART	**1.21 (0.99 – 1.65)**	**0.22**	1.03 (0.99 – 1.06)	0.10
Adherence to ART				
Good adherence	Ref.		-	-
Poor adherence	1.15 (0.59 – 1.73)	0.96		
Alcohol use				
No	Ref.		Ref.	
Yes	**0.50 (0.32 – 0.79)**	**0.003**	**0.46 (0.30 – 0.73)**	**0.001**
Presence of comorbidities				
No	Ref.		Ref.	
Yes	**0.74 (0.50 – 1.12)**	**0.15**	0.82 (0.55 – 1.22)	0.31
Medication use (HTN, diabetes, dyslipidemia) (n,%)				
No	Ref.			
Yes	0.84 (0.42 – 1.70)	0.62		
Lifestyle modification (n,%)				
No	Ref.		-	-
Yes	0.95 (0.67 -0.74)	0.77		
Family history of CVD (n,%)				
No	Ref.		-	-
Yes	1.04 (0.68 – 1.61)	0.85		

Table 2: Association between CVHI score and self-reported health status

	Coefficient (95% CI)	p-value
Model 1: Unadjusted model	0.02 (-0.47 – 0.51)	0.91
Model 2: Adjusted for socio-demographic characteristics	-0.03 (-0.05 – 0.03)	0.92
Model 3: Adjusted for insurance status and alcohol use	-0.12 (-0.81 – 0.57)	0.67
Model 4: Adjusted for other comorbidities, medication use and lifestyle modification	-0.17 (-0.88 – 0.54)	0.57

Table 3: Association between CVHI score and health status after multiple imputation of the CVHi score

	Coefficient (95% CI)	p-value
Model 1: Unadjusted model	-0.09 (-0.39 – 0.22)	0.49
Model 2: Adjusted for socio-demographic characteristics	0.001 (-0.20 – 0.21)	0.99
Model 3: Adjusted for insurance status and alcohol use	-0.006 (-0.17 – 0.16)	0.93
Model 4: Adjusted for other comorbidities, medication use and lifestyle modification	-0.02 (-0.20 – 0.15)	0.75
